# Planned organ preservation for elderly patients with rectal cancer using short course radiotherapy and a contact brachytherapy boost-an International multi-institution analysis

**DOI:** 10.1016/j.ctro.2023.100580

**Published:** 2023-01-11

**Authors:** Jacqueline Steinke, Chloe Jordan, Savvas Rossides, Helen Minnaar, Jimmy Yu, Adrian Franklin, Tim Rockall, Amandeep Singh Dhadda, Iain Andrew Hunter, Jamie Mills, Eliot Chadwick, Rafael Silverman, Joakim Folkesson, Calin Radu, Arthur Sun Myint, Alexandra J. Stewart

**Affiliations:** a St Luke’s Cancer Centre, Royal Surrey County Hospital, Guildford, England, United Kingdom; b University of Surrey, Guildford, England, United Kingdom; c Queens Centre for Oncology and Haematology, Castle Hill Hospital, Hull, England, United Kingdom; d Nottingham City Hospital, Nottingham, England, United Kingdom; e Uppsala University Hospital, Akademiska sjukhuset S-751 85, Uppsala, Sweden; f Clatterbridge Cancer Centre, Liverpool, England, United Kingdom; g University of Liverpool, Liverpool, England, United Kingdom

**Keywords:** Contact X-ray brachytherapy, Organ Preservation, Papillon technique, Rectal cancer, Short course radiotherapy

## Abstract

**Background and purpose:**

The use of external beam radiotherapy (EBRT) and contact X-Ray brachytherapy (CXB) is emerging as an effective alternative in patients with early stage rectal cancer with the intent of organ preservation (OP). Short course radiotherapy (SCRT) is an alternative EBRT schedule for patients not fit for chemotherapy or for longer courses of EBRT. There are no multicentre studies that have reported on the outcomes of SCRT with a CXB boost, therefore we present these from patients from centres from the UK and Sweden.

**Materials and methods:**

From the Guildford Colorectal Database or local databases, 258 patients who underwent SCRT and CXB with the intent of OP from five centres treated between 2007 and 2019 were identified. Response and survival data was analysed and presented.

**Results:**

With a median age of 81, 226 patients were treated with radiotherapy alone (RTA) and 32 immediately after local excision (ILE). Median follow-up was 24 months. 70% and 97% of patients in the RTA and ILE groups respectively had a complete clinical response (cCR) after SCRT with CXB. Of those, local relapse was seen in 16% of the RTA and 3% of the ILE group. Median survival was 40 months after CXB in the RTA and 52 months in the ILE group. 94% of patients remained stoma-free to the point of latest follow-up.

**Conclusion:**

This data suggests that CXB when combined with SCRT, in a mainly elderly and comorbid population, provides good palliation with stoma-avoidance. Oncological outcomes compare with previously published work. A greater focus is required on quality of life outcomes after OP.

## Highlights

• Outcomes of 258 patients from 5 centres treated with SCRT and CXB are presented.
• Median follow up was 24 months and median age 81.
• 226 patients treated with radiotherapy alone had cCR of 78% with 16% local relapse (LR).
• 32 patients treated after local excision (LE) had cCR rate of 97% with 3% LR.
• 94% of patients remained stoma-free.

## Introduction

Colorectal cancer is the 4th most common malignancy in England with an incidence of 34,825 in 2017 [1]. Just under one third of these were rectal cancers. Colorectal cancer incidence is strongly related to age, with the highest rates being amongst 85 to 89 year olds [2]. Total mesorectal excision (TME) remains the gold standard treatment for rectal cancer with the lowest risk of local recurrence. However, external beam radiotherapy (EBRT) with the addition of chemotherapy for patients who are medically fit has an established role in the management of rectal cancer, offered to patients in the neoadjuvant setting to achieve downstaging, or in those who wish to attempt to pursue an organ preservation approach [3], [4], [5].

In a large *meta*-analysis examining watch and wait outcomes in rectal cancer patients after neoadjuvant EBRT in 2018, complete clinical response (cCR) rate was estimated at 22.4 % (95 % CI 14.3–31.8), and of 692 pooled patients tumour regrowth was reported in 22.1 % of these patients, 11.7 % and 18.2 % at 1 and 2 years respectively, of which 88.4 % were successfully salvaged with surgery [6] Renehan et al. in the OnCoRe study identified 129 patients who achieved cCR after neoadjuvant radiotherapy and were subsequently managed using a watch and wait approach. 34 % of patients experienced local re-growth, the majority of whom underwent successful salvage surgery. In matched analyses between surgical resection and watch and wait patients, no differences in 3-year disease-free survival (excluding regrowth) were noted between the groups. In addition, the watch and wait group had better colostomy-free survival [7]. Myint et al. reviewed the outcomes from 83 rectal cancer patients treated with a contact X-ray brachytherapy (CXB) boost for residual tumour ≤ 3 cm after external beam radiation therapy (EBRT). Of these 83 patients, 53 (63.8 %) achieved a cCR. The data suggests that a CXB boost for patients with suspicious residual disease after EBRT can be offered as an alternative to radical surgery. In this study, patients with a sustained cCR had a low rate of local re-growth and those that had non-metastatic regrowth could be salvaged successfully [8].

Elderly patients often also present with co-morbidities that put them at higher operative risk, therefore non-surgical treatments may be preferable. In a large analysis of over 28,000 colorectal surgical patients Faiz et al. demonstrated age to be an independent risk factor for both 30-day and 365-day mortality after elective colonic resection, which was more pronounced in distal colonic as compared to proximal colonic resections [9]. Additionally, advanced age is associated with higher rates of permanent stoma, often due to non-reversal of diverting ileostomy, predominantly after anterior resection [10]. Decision analytic modelling by Smith et al. demonstrated that using an organ preservation approach in fit 80-year-olds may offer a 10 % survival benefit at one year [11]. However, older patients may not be fit enough to undergo five to six weeks of EBRT with concomitant chemotherapy (CRT). Short course radiotherapy (SCRT) 25 Gray in 5 fractions is an established and effective treatment regime in the pre-operative setting [12]. In this setting SCRT has been compared to CRT and has not shown a statistical difference in local recurrence or overall survival [13], [14].

Ten-year results of the Lyon R 96–02 trial published in 2011 used an EBRT dose of 39 Gray in 13 fractions in both control and CXB boost arms, with 10 year local recurrence rates of 10–15 %[15]. Some data is available from single institution outcomes that have included patients treated with either CRT, long course EBRT or SCRT and CXB as part of an organ-sparing approach [16], [17], [18]. However, there is no published data reporting outcomes only in this group of patients. Therefore, the clinical outcomes for patients undergoing SCRT and CXB from five institutions for the treatment of rectal cancer in mainly elderly or medically unfit patients is presented.

## Methods

### Patient selection

A search was performed using the Guildford Colorectal database to identify patients who underwent CXB and SCRT. Centres also used prospectively-maintained local databases to provide information on patients treated before inception of the Guildford Colorectal database or to add any missing data that had not been entered into the national database. Two hundred and fifty-eight consecutive patients who received SCRT and CXB were included in the analysis. This included those who had CXB with SCRT as primary treatment, the radiotherapy alone group (RTA) and those who had these immediately after local excision (ILE). Patients treated with CXB for regrowth after primary EBRT were excluded from original data collection and four patients treated for regrowth after local excision were excluded after data collection.

The Colorectal Database was developed to collect clinical outcome data on patients undergoing CXB for rectal cancer. The database is currently in use by four centres in the UK: Royal Surrey Hospital Guildford, Clatterbridge Cancer Centre Liverpool, Nottingham City Hospital and Queens Oncology Centre Hull and by Uppsala Hospital, Sweden. All patients have their data pseudonymised and entered into the colorectal database prospectively. The database includes patient characteristics, tumour characteristics and staging, treatment intent, and follow-up data. Data was collected from the inception of the database (April 2014) or inception of the CXB service, whichever was later, until the end of December 2019. Data from patients treated prior to database inception was collated from local databases for the purpose of this analysis.

Baseline tumour characteristics and staging were assessed using clinical examination, endoscopic evaluation and computed tomography (CT) and magnetic resonance imaging (MRI) scanning. MRI was used to determine T and N stage, in the absence of contraindications. Patients received CXB in bi-weekly fractions according to staging and risk factors. EBRT was delivered with 3D-conformal or Intensity Modulated Radiation Therapy (IMRT) delivering 25 Gy in 5 fractions over a period of one week. EBRT was given prior to CXB if the tumour size was over 3 cm at presentation.

An assessment of medical fitness for surgery or operability was made in each case at the time of referral, based primarily on number and extent of comorbidities, and ECOG performance status. Patients who were potential surgical candidates who had opted for a non-surgical approach due to age or surgical risk-factors were closely followed-up, with regular MRI, digital rectal examination (DRE), and flexible sigmoidoscopy, every-three months during the first two years, and six-monthly during year three. CT scanning of the abdomen and pelvis was performed according to local follow-up protocols. For patients who were not surgical candidates a baseline MRI and sigmoidoscopy was recommended at 3 months to assess treatment response. The follow-up of each patient was recorded in the database.

### Statistical analysis

Follow-up duration was calculated from the commencement of radiotherapy treatment (either SCRT or CXB, whichever was first) until the last known date of follow-up or death. Statistical Analysis was performed using Microsoft Excel, GraphPad Prism Version 8.4.3 and SPSS version 22.

Categorical variables were compared according to treatment pathway using a Chi-Squared test. Continuous variables were compared using an independent *t*-test for parametric and Mann-Whitney-U for non-parametric data. Uni- and multivariate analysis was undertaken with multiple logistic regression to examine the effect of factors on complete response and Cox regression analysis for local relapse and disease free survival.

The Kaplan–Meier methodology was used to calculate overall survival (OS) and disease-free survival (DFS) rates at 2, 3 and 5 years and median survival where applicable. Relapse was defined as local if within the rectum, regional if within the pelvis and distant if beyond the pelvis, meaning locoregional relapse would be in-field and distant out-out-field of EBRT.

Disease-free survival was defined as survival with complete response without relapse (either locoregional or distant metastatic disease) or either incomplete response or locoregional relapse with successful salvage treatment (usually excisional surgery). Patients were censored at the point of death, non-salvaged disease or last known follow-up. Comparison of Kaplan-Meier survival curves was performed using the Logrank test. Subgroup analysis of the RTA group was undertaken, comparing those that underwent EBRT first (n = 122) and those who underwent CXB first (n = 103), as it was hypothesised that a longer interval between CXB and EBRT treatment may have an impact on outcomes. Within this subgroup analysis local relapse-free survival (LRFS) was defined as survival without local relapse, (death was not included as an event).

## Results

### Clinical and tumour characteristics

258 patients underwent SCRT and CXB between 2007 and 2019. This included 117 patients from Clatterbridge Cancer Centre Liverpool, 36 patients from Castle Hill Hospital Hull, 27 patients from City Hospital Nottingham, 44 patients from Uppsala, Sweden and 34 from The Royal Surrey Guildford. The median age was 81 years (range 49–103). Only 27.5 % of patients overall were considered medically operable at the time of referral, 39.1 % and 33.3 % were classed as high surgical risk and medically unfit respectively. Five patients were unable to be staged by MRI (four had no T or N stage and one no N stage) due to contraindications, and nearly-two thirds (63.4 %) of patients treated were T2.

The majority of patients underwent radiotherapy alone, RTA (226 patients) with 32 undergoing this immediately after LE (ILE). Other patient characteristics and treatment given broken down by treatment pathway are shown in Table 1.

**Table 1:** Baseline characteristics for 258 patients in RTA and ILE groups.

		**Total (n = 258)**		**Radiotherapy Alone (n = 226)**		**Immediately post LE (n = 32)**		**p value**
		**no.**	**%**	**no.**	**%**	**no.**	**%**	
*Patient characteristics*								
**Age**	(median, range)	81 (49–103)		82 (49–103)		79 (55–94)		0.003
	< 75	63	24.4	50	22.1	13	40.6	0.02
	75–84	103	39.9	89	39.4	14	43.8	
	> 85	92	35.7	87	38.5	5	15.6	
**Gender**	Male	178	69.0	155	68.6	23	71.9	0.71
	Female	80	31.0	71	31.4	9	28.1	
**Operability (medical)**	Operable	71	27.5	51	22.6	20	62.5	<0.001
	High risk	101	39.1	90	39.8	11	34.4	
	Medically unfit for surgery	86	33.3	85	37.6	1	3.1	

*Tumour Characteristics*								
T stage*	T1 − 2	182	71.7	151	68.0	31	96.9	0.001
	T3 − 4	72	28.3	71	32.0	1	3.1	
	*T1*	*21*	*8.3*	*5*	*2.2*	*16*	*50.0*	
	*T2*	*161*	*63.4*	*146*	*65.8*	*15*	*46.9*	
	*T3*	*67*	*26.4*	*66*	*29.7*	*1*	*3.1*	
	*T4*	*5*	*2.0*	*5*	*2.3*	*0*	*0.0*	
N stage**	N0	203	80.2	171	77.4	32	100.0	0.003
	N1 or N2	50	19.8	50	22.6	0	0.0	

*Treatment Characteristics*								
Time interval***	(median, range)	57 (4–345)		57 (4–345)		58 (24–190)		0.98
**CXB dose**	30–60	37	14.3	8	3.5	29	90.6	<0.001
	80–90	155	60.0	152	67.3	3	9.4	
	110–120	66	25.6	66	29.2	0	0.0	
**Fractionation**	30 Gy in 1 Fr	2	0.8	2	0.9	0	0.0	
	60 Gy in 2 Fr	35	13.6	6	2.7	29	90.6	
	80 Gy in 3 Fr	7	2.7	7	3.1	0	0.0	
	90 Gy in 3 Fr	148	57.4	145	64.2	3	9.4	
	110 Gy in 4 Fr	48	18.6	48	21.2	0	0.0	
	120 Gy in 4 Fr	18	7.0	18	8.0	0	0.0	

*4 patients had no T stage determined ** 5 patients had no N stage determined *** Time interval was defined as time interval in terms of days between CXB treatment and external beam radiotherapy.

The median age of the ILE group was slightly lower (79 vs 82 years, p = 0.003), and patients were more likely to be staged as T1/T2 vs T3/T4 (97 % vs 68 %, p = 0.001) and classed as medically operable (63 % vs 23 %, p < 0.001) compared to the RTA group.

## Follow-up, treatment given and outcomes

Median follow-up for all patients was 24.0 months (range 2–114.9 months), which differed by treatment pathway (25.7 for RTA, 18.0 for ILE, p = 0.006). All patients received 25 Gy in 5 fractions of EBRT alongside CXB, of which 54 % had EBRT and 46 % CXB first. Total CXB doses ranged from 30 Gy to 120 Gy. Median dose was 90 Gy for the RTA and 60 Gy for the ILE group. CXB doses administered differed significantly between the two treatment pathways, in keeping with recommendations in published guidelines [19].

Of the combined cohort, in those who had response assessment (240/258 patients) 73.8 % had a cCR, 6.7 % had a near complete response with 12.9 % showing partial response and 6.7 % having persistent disease. A statistically significant increased cCR or near cCR rate was seen in the ILE group when compared to the RTA (96.9 vs 77.9 %, p = 0.01). Of those with cCR, 16.0 % of the RTA group had later local relapse and 3.2 % of the ILE group (p < 0.001). Only 2.7 % of all patients had metastatic disease detected during the follow-up period, which did not differ by treatment pathway (3.1 % ILE and 2.7 % RTA, p = 1.0). Permanent stoma rate for the whole cohort was 6.2 %, which did not differ significantly between the two groups. A further breakdown of treatment given, follow-up and outcomes is shown in Table 2.

**Table 2:** Follow-up and Outcomes of 258 patients in RTA and ILE groups.

		**Total (n = 258)**		**Radiotherapy Alone (n = 226)**		**Immediately post LE (n = 32)**		**p value**
		**no.**	**%**	**no.**	**%**	**no.**	**%**	
*Follow up*								
Follow up *	(median, range)	24.0 (2–114.9)		25.7 (2–114.9)		18.0 (4.0–62.8)		0.006
**Status at latest F/U**	Dead	126	49.6	121	54.5	5	15.6	<0.001
	Alive	128	50.4	101	45.5	27	84.4	
	*-With no recurrence*	*103*	*40.6*	*77*	*34.7*	*26*	*81.3*	
	*-With treated recurrence*	*7*	*2.8*	*7*	*3.2*	*0*	*–*	
	*-With disease*	*18*	*7.1*	*17*	*7.7*	*1*	*3.1*	

*Outcomes*								
Clinical Response **	Partial response or persistent disease	47	19.6	46	22.1	1	3.1	0.01
	Complete or near response	193	80.4	162	77.9	31	96.9	
	*Complete*	*177*	*73.8*	*146*	*70.2*	*31*	*96.9*	
	*Near*	*16*	*6.7*	*16*	*7.7*	*0*	*0.0*	
	*Partial*	*31*	*12.9*	*30*	*14.4*	*1*	*3.1*	
	*Persistent*	*16*	*6.7*	*16*	*7.7*	*0*	*0.0*	
	*Not Assessed/ Unknown*	*18*	*–*	*18*	*–*	*0*	*–*	
Local relapse***	Absent	166	86.0	136	84.0	30	96.8	<0.001
	Present	27	14.0	26	16.0	1	3.2	
**Time to Local Relapse**	(median, range)	14.2 (9–50)		14 (9–50)		24		0.46
**Distant Metastases**	Absent	251	97.3	220	97.4	31	96.9	1.00
	Present	7	2.7	6	2.7	1	3.1	
**Stoma**	None	242	93.8	211	93.4	31	96.9	0.70
	Permanent stoma	16	6.2	15	6.6	1	3.1	

**Percentages calculated of those with response assessment, ***Local relapse calculated as % of those with complete or near response, Time to local relapse and follow up was calculated in months.

## Survival analysis

**Fig. 1:**
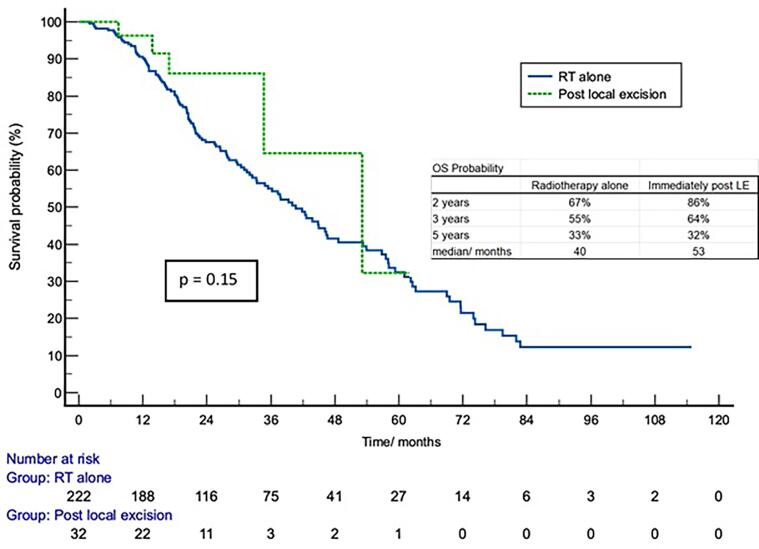
Kaplan Meier Survival Curves for OS comparing RTA and ILE groups.

**Fig. 2:**
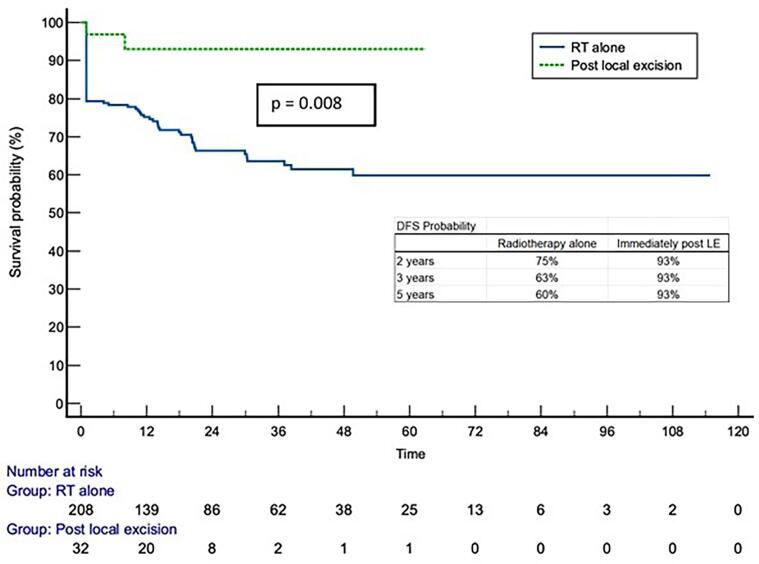
Kaplan Meier Survival Curves for DFS comparing RTA and ILE groups.

Kaplan Meier survival curves of overall survival (OS) and disease-free survival (DFS) for RTA and ILE cohorts are shown in Fig. 1, Fig. 2. There was a statistically significant difference in DFS when survival curves for RTA and ILE groups were compared (p = 0.008). No difference was shown for OS (p = 0.15). Survival probabilities for the two groups at 2, 3 and 5 years are shown in tables on the respective survival curves. Median survival was 40 and 53 months from date of commencement of treatment in the RTA and ILE groups respectively with median age at death 86 years (range 61–100 years) for the RTA and 84 years (range 60–87 years) for the ILE group.

## Subgroup analysis of the radiotherapy alone group

Subgroup analysis of the RTA group comparing those who underwent EBRT first and CXB first was undertaken. One patient was excluded from the analysis due to missing data, giving a cohort of 225 in the analysis, 103 who had CXB first and 122 who had EBRT first. There were baseline differences in the two groups, which would be expected according to treatment protocol which dictates larger, more advanced stage tumours be treated by up front EBRT. Age, gender and CXB doses administered did not differ significantly, but there was a higher proportion of T1/T2 vs T3/4 in the CXB first group (81.6 vs 55.9 %, p < 0.001), higher proportion of nodal positivity (N1/2) vs N0 stage (29.9 vs 14.5 %, p = 0.01) and lower rates of medical operability (17.2 vs 29.1 %, p = 0.002) in the EBRT first group.

Mean time interval between the CXB and EBRT was significantly longer in the EBRT first group, at 98.4 days vs 51.2 days in the CXB first group, p < 0.001. Of those who underwent response assessment (207/225 patients), a higher complete or near response rate was seen in the CXB first group (84.8 %) vs the EBRT first (72.2 %), p = 0.03.

## Analysis of complete response

A chi-square test of independence comparing the two groups showed that there was a significant association between treatment pathway and response, χ2 (1, N = 207) = 4.84, p = 0.03. Univariate logistic regression analysis was conducted on the overall cohort of 207 patients who had response assessment, which suggested a more advanced T stage (T3/4 vs T1/2) and a CXB dose of 110 or 120 Gy compared to ≤ 90 Gy was associated with a decreased chance of complete or near complete response. Only CXB dose was shown to be significant on multivariate analysis (OR 0.42, 95 % CI 0.20–0.89, p = 0.02). All other factors including age, gender, nodal stage, time interval between treatments and medical operability showed no significant association. Subgroup analysis of the EBRT first group (108 patients) showed that on univariate analysis being classed as medically unfit for surgery (vs high risk or operable) and a higher CXB dose was associated with a decreased chance of complete or near response. On multivariate analysis these, as well as female gender, were associated with a lower chance of complete response. No factors were shown to affect rates of complete response in the CXB first subgroup analysis. Results of these analyses are shown in supplementary Tables 1 and 2.

## Analysis of disease-free survival

**Fig. 3:**
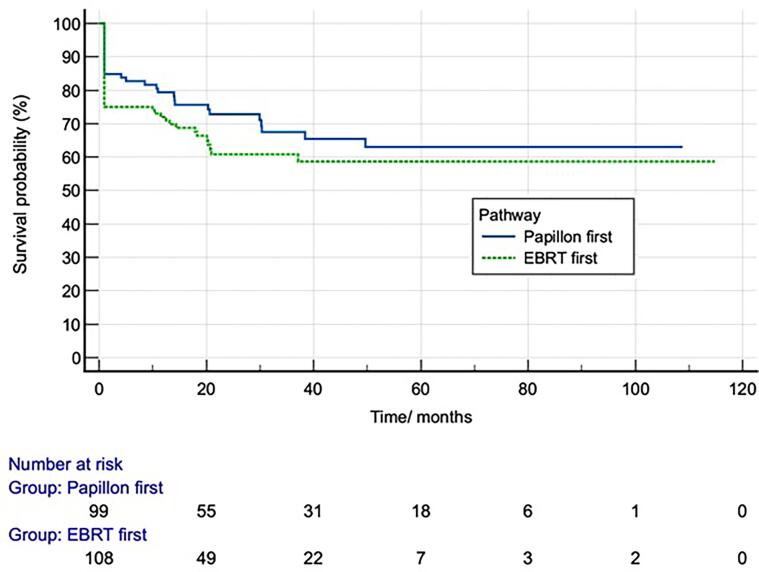
Kaplan Meier Survival Curves for DFS of 207 RTA patients with response assessment stratified by treatment pathway.

Survival analysis of the 207 patients who underwent response assessment was undertaken and stratified by treatment pathway. The graph of this is shown in Fig. 3. There was no significant difference in survival curves seen (Logrank, p = 0.22). Cox regression analysis was performed to examine the effects of variables on disease free survival. In the overall cohort uni- and multivariate analysis showed that T3/T4 vs T1/2 stage was associated with a lower chance of disease-free survival. In the EBRT first subgroup univariate analysis CXB dose was significantly associated with a lower chance of DFS and on multivariate this was again shown, as well as operability. Although in the CXB first group time interval showed a statistically significant association, the hazards ratio was so close to 1 not to be clinically significant. Results of these analyses are shown in supplementary Tables 3 and 4.

## Analysis of local relapse

**Fig. 4:**
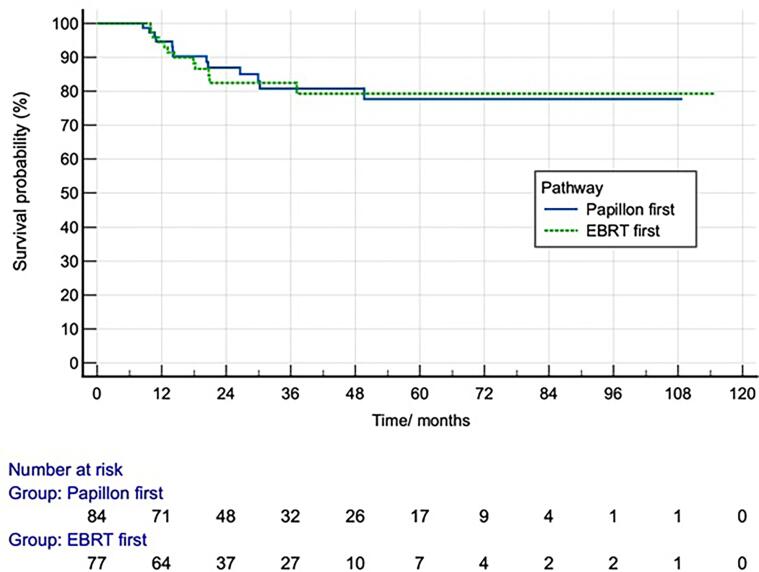
Kaplan Meier Survival Curves for Local-relapse free survival of 161 RTA patients with near or complete response stratified by treatment pathway.

Survival analysis of local-relapse free survival stratified according to treatment pathway of the 161 patients with complete or near response is shown in Fig. 4. Logrank test comparing survival curves of the two groups showed no significant difference (p = 0.87). Two- and five-year local relapse-free survival was 85 % and 78 % respectively. Uni- and multivariate Cox regression analysis was performed to examine the effects of age, time interval between CXB and external beam radiotherapy, gender and T stage, N stage, medical operability and CXB dose on local relapse. This showed that none of the factors were significantly associated with local relapse.

## Discussion

These results show that of 258 patients who underwent SCRT with CXB either alone or combined with LE, the median age was 81 years and 72 % were considered either high risk or medically unfit for surgery. Median follow-up was 24 months and the majority of patients overall were T2, although 50 % of the ILE group were T1. Of those with response assessment, 80.4 % had a complete or near complete clinical response and 14 % had local relapse, at a median of 14 months from treatment. Higher administered CXB doses, medical unfitness for surgery and more advanced *T*-stage were shown to be negative prognostic factors for complete response and disease-free survival.

The majority of patients did not undergo further excisional surgery, and only 16 (6.2 %) required permanent stoma formation. Those undergoing CXB with SCRT immediately after local excision had higher disease-free survival when compared to those who had it as sole treatment (93 vs 63 % at 3 years). Higher rates of cCR and lower rates of local recurrence were seen in the ILE cohort, though this could be due to the higher proportion of T1 tumours at diagnosis. The median age at death was 86, above the UK life expectancy, at 82.3 for males and 85.3 for females [20].

In a cohort of 148 Swedish patients referred into a watch and wait programme treated with CRT or SCRT neoadjuvantly for rectal cancer, 88 were deemed to fulfil the criteria for cCR. Of these 44 had undergone SCRT. These had a much younger median age than the current study, 68 years versus 81 years, and a lower proportion were T1/2, 52 % versus 80 %. Only 14 % experienced local regrowth at a median interval of 40 weeks, which is a faster time to relapse than seen in our study [21].

Although there are no published series of a cohort undergoing only SCRT with a CXB boost, multiple previously published series and trials have reported on outcomes in patients undergoing EBRT with CXB with the intent of organ preservation. Of recent series reporting outcomes after EBRT with CXB, cCR rates ranged from 72 to 92 % with local regrowth rates ranging from 10 to 31 % [8], [17], [18], [22]. Disease-free survival where reported has ranged from 88 % at 3 years to 53 % at 5 years. With the exception of the Lyon R 96–02 randomised trial [22], where a total dose of 39 Gy in 13 fractions was given, the majority of patients in these series received CRT alongside CXB and the median age was lower than in our cohort with higher rates of operable patients. A Dutch study examined short-term outcomes in 19 older or inoperable patients with a median age of 80 treated with contact radiotherapy, of whom 58 % were staged as T3, and 32 % had additional CRT, 43 % had SCRT 25 Gy in 5 fractions or 39 Gy in 13 fractions and 21 % had prior local excision. With median follow-up of 13 months, local control of the tumour was attained in 68 % of patients, with a cCR rate of 47 %. In those who attained local control no regrowths were reported to the point of follow-up [23] in comparison to 80 % complete clinical response with a local regrowth of 14 % in the current series.

The GRECCAR2 randomised trial reported outcomes in surgically fit patients undergoing CRT who had a cCR and were randomised to either LE or TME surgery. The LE arm, with a median age of 61 showed a 5-year local recurrence rate of 7 % [24]. The CONTEM1 study was a multi-centre cohort study looking at outcomes of 194 patients with a median age of 69, treated with LE and adjuvant CXB, of which 14 % received additional SCRT, 18 % long course radiotherapy alone and 55 % CRT. With a median follow-up of 77 months, local recurrence rates were 7.7 % with a distant metastatic rate of 9.3 %[25]. Overall survival was higher than in our cohort, at 81 % at 6 years, which may be explained by the lower age and higher rates of operability than in our post LE group as well as the higher doses of EBRT given.

A single UK centre cohort study published by Smith et al. in 2019 looked at outcomes following LE with CXB and reported on 180 patients with a median age of 70, 82 % of whom were performance status 0 or 1. With a median follow-up of 36 months, 94 % were free of local recurrence and 94 % were stoma-free [26]. These studies with higher median follow-up time and larger numbers of patients, mainly report on outcomes of younger and surgically fit patients, where treatment with palliative intent was excluded, in contrast to our study. Despite this, our local recurrence rate of 3 % compares favourably with the above cohorts, suggesting that LE when combined with SCRT and CXB provides good oncological control and high chances of organ preservation in a less fit patient population. It is important to note that some patients included in CONTEM1 and Smith et al. study were also included in the current analysis.

This was retrospective observational study of a prospectively-maintained database. Being a multi-centre study, input into the database was centre-dependent and there will be have been variations in how the data was collected and interpreted. Although data was complete for most study parameters, there was a significant proportion of missing data in the cause of death, which meant disease-specific survival was unable to be confidently calculated. This study focuses on survival and outcomes rather than quality of life or functional data, which are important to consider, especially in elderly, frail or comorbid patients. Further research, including the CITRuS trial examining patient reported outcomes following CXB will seek to address this (ClinicalTrials.gov Identifier: NCT04697394) [27].

## Conclusions

This is the first multi-centre study to report on outcomes of patients undergoing SCRT with a CXB boost. The cohort of patients undergoing this treatment was mainly elderly and high risk or unfit for major surgery, but nevertheless good rates of cCR have been demonstrated. This is particularly so for the cohort who had first undergone LE, although this group was a favourable prognostic group at the outset, defining themselves as medically fit for general anaesthesia for local excision and 50 % being T1. CXB is generally a well-tolerated treatment option with low levels of toxicity and can provide excellent palliation in symptomatic low rectal tumours and our results support the use of it in this setting.

## Declaration of Competing Interest

The authors declare that they have no known competing financial interests or personal relationships that could have appeared to influence the work reported in this paper.
