# Supplementary material for: Planned organ preservation for elderly patients with rectal cancer using short course radiotherapy and a contact brachytherapy boost-an International multi-institution analysis
Source: Clin Transl Radiat Oncol. 2023 Jan 11;39:100580. doi: 10.1016/j.ctro.2023.100580 (PMC9852541; doi:10.1016/j.ctro.2023.100580)
Supplement: Supplementary data 1 [file mmc1.docx]

**Supplementary Table 1 - Univariate analysis for 207 patients on complete response, according to treatment pathway**

|  |  | **Overall (n = 207)** | | | **Papillon first (n = 99)** | | | **EBRT first (n = 108)** | | |
| --- | --- | --- | --- | --- | --- | --- | --- | --- | --- | --- |
|  |  | **OR** | **95% CI** | **p value** | **OR** | **95% CI** | **p value** | **OR** | **95% CI** | **p value** |
| **Age** | as continuous variable | 0.99 | 0.95 - 1.03 | 0.68 | 0.94 | 0.86 - 1.02 | 0.12 | 1.01 | 0.97 - 1.06 | 0.63 |
|  | < 75 years old | Ref | | | Ref | | | Ref | | |
|  | 75 - 84 years old | 1.08 | 0.44 - 2.63 | 0.87 | 0.28 | 0.03 - 2.53 | 0.26 | 1.53 | 0.51 - 4.54 | 0.45 |
|  | > 85 years old | 0.77 | 0.33 - 1.82 | 0.55 | 0.19 | 0.02 - 1.62 | 0.13 | 1.18 | 0.42 - 3.34 | 0.75 |
| **Time interval** | as continuous variable | 1.00 | 0.99 - 1.01 | 0.79 | 1.01 | 0.98 - 1.04 | 0.40 | 1.00 | 0.99 - 1.01 | 0.60 |
|  | < 57 days | Ref | | | Ref | | | Ref | | |
|  | ≥ 57 days | 0.65 | 0.33 - 1.26 | 0.20 | 0.75 | 0.21 - 2.64 | 0.65 | 1.05 | 0.41 - 2.74 | 0.91 |
| **Gender** | male | Ref | | | Ref | | | Ref | | |
|  | female | 0.51 | 0.26 - 1.01 | 0.05 | 0.75 | 0.23 - 2.44 | 0.64 | 0.42 | 0.18 - 1.01 | 0.05 |
| **T stage** | T1 or T2 | Ref | | | Ref | | | Ref | | |
|  | T3 or T4 | 0.47 | 0.24 - 0.94 | 0.03 | 0.55 | 0.15 - 1.98 | 0.36 | 0.57 | 0.24 - 1.34 | 0.20 |
| **N Stage** | N0 | Ref | | | Ref | | | Ref | | |
|  | N1 or N2 | 0.67 | 0.31 - 1.44 | 0.30 | 1.08 | 0.22 - 5.42 | 0.92 | 0.68 | 0.27 - 1.70 | 0.41 |
| **Operability** | Operable or High risk | Ref | | | Ref | | | Ref | | |
|  | Inoperable | 0.65 | 0.34 - 1.28 | 0.21 | 1.24 | 0.40 - 3.79 | 0.71 | 0.37 | 0.15 - 0.88 | 0.03 |
| **Papillon dose** | ≤ 90 | Ref | | | Ref | | | Ref | | |
|  | 110 - 120 | 0.46 | 0.23 - 0.92 | 0.03 | 0.80 | 0.23 - 2.82 | 0.73 | 0.39 | 0.16 - 0.94 | 0.04 |

**Supplementary Table 2- Multivariate analysis for 207 patients on complete response, according to treatment pathway**

|  | **Overall (n = 207)** | | | **Papillon first (n = 99)** | | | **EBRT first (n = 108)** | | |
| --- | --- | --- | --- | --- | --- | --- | --- | --- | --- |
|  | **OR** | **95% CI** | **p value** | **OR** | **95% CI** | **p value** | **OR** | **95% CI** | **p value** |
| **Age** | 1.00 | 0.96 - 1.04 | 0.98 | 0.94 | 0.86 - 1.02 | 0.15 | 1.01 | 0.96 - 1.07 | 0.66 |
| **Time interval between Papillon and EBRT** | 1.00 | 0.99 - 1.01 | 0.98 | 1.01 | 0.99 - 1.04 | 0.35 | 1.00 | 0.99 - 1.01 | 0.85 |
| **Gender (male vs female)** | 0.52 | 0.26 - 1.07 | 0.08 | 0.69 | 0.20 - 2.38 | 0.56 | 0.34 | 0.12 - 0.93 | 0.04 |
| **T stage (T1/2 vs T3/4)** | 0.49 | 0.22 - 1.07 | 0.07 | 0.73 | 0.16 - 3.46 | 0.69 | 0.71 | 0.25 - 2.02 | 0.52 |
| **N stage (N0 vs N1/2)** | 0.99 | 0.41 - 2.38 | 0.97 | 1.25 | 0.19 - 8.05 | 0.82 | 0.80 | 0.26 - 2.46 | 0.70 |
| **Operability (Operable or high risk vs Inoperable)** | 0.60 | 0.28 - 1.26 | 0.18 | 1.44 | 0.44 - 4.71 | 0.54 | 0.26 | 0.09 - 0.78 | 0.02 |
| **Papillon dose (≤ 90 vs 110-120)** | 0.42 | 0.20 - 0.89 | 0.02 | 0.81 | 0.21 - 3.06 | 0.75 | 0.26 | 0.09 - 0.72 | 0.01 |

|  |  | **Overall (n = 207)** | | | **Papillon first (n = 99)** | | | **EBRT first (n = 108)** | | |
| --- | --- | --- | --- | --- | --- | --- | --- | --- | --- | --- |
|  |  | **HR** | **95% CI** | **p value** | **HR** | **95% CI** | **p value** | **HR** | **95% CI** | **p value** |
| **Age** | as continuous variable | 1.01 | 0.98 - 1.04 | 0.42 | 1.03 | 0.98 - 1.08 | 0.22 | 1.00 | 0.97 - 1.03 | 0.97 |
|  | < 75 years old | Ref | | | Ref | | | Ref | | |
|  | 75 - 84 years old | 1.29 | 0.68 - 2.46 | 0.44 | 1.76 | 0.57 - 5.45 | 0.33 | 1.14 | 0.51 - 2.53 | 0.75 |
|  | > 85 years old | 1.37 | 0.72 - 2.60 | 0.33 | 2.19 | 0.72 - 6.68 | 0.17 | 1.03 | 0.46 - 2.29 | 0.95 |
| **Time interval** | as continuous variable | 1.00 | 1.00 - 1.00 | 0.96 | 0.97 | 0.95 - 1.00 | 0.03 | 1.00 | 1.00 - 1.01 | 0.73 |
|  | < 57 days | Ref | | | Ref | | | Ref | | |
|  | ≥ 57 days | 1.27 | 0.79 - 2.04 | 0.32 | 0.78 | 0.30 - 2.05 | 0.62 | 1.47 | 0.68 - 3.19 | 0.33 |
| **Gender** | male | Ref | | | Ref | | | Ref | | |
|  | female | 1.20 | 0.73 - 1.96 | 0.47 | 1.00 | 0.46 - 2.19 | 1.00 | 1.33 | 0.70 - 2.52 | 0.38 |
| **T stage** | T1 or T2 | Ref | | | Ref | | | Ref | | |
|  | T3 or T4 | 1.79 | 1.11 - 2.88 | 0.02 | 1.57 | 0.70 - 3.52 | 0.28 | 1.83 | 0.97 - 3.45 | 0.06 |
| **N stage** | N0 | Ref | | | Ref | | | Ref | | |
|  | N1 or N2 | 1.25 | 0.73 - 2.14 | 0.41 | 1.02 | 0.39 - 2.68 | 0.97 | 1.26 | 0.65 - 2.46 | 0.49 |
| **Operability** | Operable or high risk | Ref | | | Ref | | | Ref | | |
|  | Inoperable | 1.42 | 0.89 - 2.27 | 0.14 | 1.18 | 0.57 - 2.41 | 0.66 | 1.77 | 0.95 - 3.30 | 0.07 |
| **Papillon dose** | ≤ 90 | Ref | | | Ref | | | Ref | | |
|  | 110 - 120 | 1.57 | 0.96 - 2.55 | 0.07 | 1.00 | 0.43 - 2.34 | 1.00 | 1.95 | 1.04 - 3.63 | 0.04 |

**Supplementary Table 3- Univariate analysis for 207 patients on disease free survival, according to treatment pathway**

**Supplementary Table 4 - Multivariate analysis for 207 patients on disease free survival, according to treatment pathway**

|  | **Overall (n = 207)** | | | **Papillon first (n = 99)** | | | **EBRT first (n = 108)** | | |
| --- | --- | --- | --- | --- | --- | --- | --- | --- | --- |
|  | **HR** | **95% CI** | **p value** | **HR** | **95% CI** | **p value** | **HR** | **95% CI** | **p value** |
| **Age** | 1.01 | 0.98 - 1.04 | 0.59 | 1.03 | 0.98 - 1.08 | 0.25 | 1.01 | 0.97 - 1.04 | 0.75 |
| **Time interval between Papillon and EBRT** | 1.00 | 1.00 - 1.01 | 0.90 | 0.97 | 0.95 - 1.00 | 0.03 | 1.00 | 1.00 - 1.01 | 0.50 |
| **Gender (male vs female)** | 1.14 | 0.69 - 1.87 | 0.62 | 1.03 | 0.46 - 2.31 | 0.95 | 1.36 | 0.68 - 2.70 | 0.38 |
| **T stage (T1/2 vs T3/4)** | 1.79 | 1.08 - 2.99 | 0.03 | 1.09 | 0.41 - 2.90 | 0.87 | 1.70 | 0.86 - 3.39 | 0.13 |
| **N stage (N0 vs N1/2)** | 0.89 | 0.49 - 1.61 | 0.70 | 0.78 | 0.25 - 2.39 | 0.66 | 0.91 | 0.43 - 1.93 | 0.80 |
| **Operability (Operable or high risk vs Inoperable)** | 1.57 | 0.94 - 2.61 | 0.08 | 1.08 | 0.51 - 2.28 | 0.83 | 2.40 | 1.17 - 4.89 | 0.02 |
| **Papillon dose (< or equal to 90 vs 110-120)** | 1.63 | 0.99 - 2.69 | 0.05 | 0.99 | 0.41 - 2.39 | 0.98 | 2.32 | 1.21 - 4.47 | 0.01 |
